# Performance of Conventional Urine Culture Compared to 16S rRNA Gene Amplicon Sequencing in Children with Suspected Urinary Tract Infection

**DOI:** 10.1128/spectrum.01861-21

**Published:** 2021-12-22

**Authors:** Christopher W. Marshall, Marcia Kurs-Lasky, Christi L. McElheny, Sophia Bridwell, Hui Liu, Nader Shaikh

**Affiliations:** a Department of Biological Sciences, Marquette University, Milwaukee, Wisconsin, USA; b University of Pittsburgh grid.21925.3dgrid.471408.e School of Medicine, Children’s Hospital of Pittsburgh, Division of General Academic Pediatrics, Pittsburgh, Pennsylvania, USA; University of Texas Southwestern Medical Center

**Keywords:** urinary tract infection, urine culture, accuracy, urobiome, 16S rRNA gene

## Abstract

Because some organisms causing urinary tract infection (UTI) may be difficult to culture, examination of bacterial gene sequences in the urine may provide a more accurate view of bacteria present during a UTI. Our objective was to estimate how often access to 16S rRNA gene amplicon sequencing alters diagnosis and/or clinical management. The study was designed as a cross-sectional study of a convenience sample of children with suspected UTI. The setting was the emergency department or outpatient clinic at six pediatric centers. Participants included children 2 months to 10 years of age suspected of UTI. We categorized the results of urine culture as follows: “likely UTI” (≥100,000 CFU/ml of a single uropathogen), “possible UTI” (10,000 to 99,000 CFU/ml of a uropathogen or ≥100,000 CFU/ml of a single uropathogen plus other growth), and “unlikely UTI” (no growth or growth of nonuropathogens). Similarly, we categorized the results of 16S rRNA gene sequencing into the same three categories using the following criteria: likely UTI (≥90% relative abundance of a uropathogen), possible UTI (50 to 89% relative abundance of a uropathogen), and unlikely UTI (remainder of samples). The main study outcome was concordance between conventional culture results and 16S rRNA gene sequencing. Concordance between the two methods was high in children with likely and unlikely UTI by conventional culture (95% and 87%, respectively). In children with possible UTI according to conventional culture, 71% had a single uropathogen at a relative abundance of ≥90% according to 16S rRNA gene sequencing data. Concordance between conventional culture and 16S rRNA gene amplicon sequencing appears to be high. In children with equivocal culture results, 16S rRNA gene results may provide information that may help clarify the diagnosis.

**IMPORTANCE** Concordance between conventional culture and 16S rRNA gene amplicon sequencing appears to be high. In children with equivocal culture results, 16S rRNA gene results may provide information that may help clarify the diagnosis.

## INTRODUCTION

It is now firmly established that conventional microbiological techniques used for clinical testing miss a significant proportion of difficult-to-culture organisms (1–3). By overcoming limitations in our ability to cultivate microorganisms, high-throughput sequencing promises to provide a more accurate view of the bacteria present during a urinary tract infection (UTI). Furthermore, 16S rRNA gene panels being developed promise to identify the causative microbial pathogen within hours of sample collection (4). Theoretically, the availability of such panels, which in the near future may also screen for genes conferring resistance to frequently used antimicrobials, could drastically change our approach to the diagnosis and treatment of UTIs. Enhanced timely detection of pathogens is particularly relevant in children not only because symptoms of UTI are less reliably reported but also because missed febrile UTIs are associated with a significant risk of permanent renal scarring (5, 6).

Among children with clear symptoms of UTI, many of whom also exhibit signs of inflammation of the urinary tract (i.e., pyuria), many are eventually found to have a negative culture. As such, clinicians often speculate whether organisms that are not easily culturable using conventional microbiological techniques could have been responsible for the observed signs and symptoms. Other children have culture results that are equivocal. These include children with a single uropathogen at low colony counts, children with several uropathogens, and children with mixed growth of both uropathogens and nonpathogens. Accordingly, we aimed to determine whether access to results from 16S rRNA gene amplicon sequencing would provide answers to these frequently encountered clinical questions.

To our knowledge only three studies (two in adults) to date have concurrently examined 16S rRNA gene amplicon sequencing and conventional urine cultures in patients being evaluated for UTI (7–9), and each of these studies included fewer than 15 patients with UTI. Therefore, to assess the utility of the technique, more studies are needed.

In this study, we calculated how frequently conventional culture obtained from hospital clinical microbiology laboratories provided results concordant with those of culture-independent 16S rRNA gene amplicon sequencing in children with suspected UTI.

## RESULTS

Table 1 describes the demographic characteristics of the 118 children with suspected UTI included in this study. The mean age of the included children was 41 months, and 90% were febrile. Of the 118 samples, 56 were collected using bladder catheterization and 62 using clean catch (Table 1). There were no demographic or clinical differences between the 118 children in this report and the 30 children who were excluded (26 children were excluded because they had <1,000 sequences per sample and 4 because they met exclusion criteria).

**TABLE 1:** Characteristics of the 118 children included

Characteristic	No. (%) unless otherwise indicated
Age (mo) at consent [mean (SD)]	40.9 (30.9)
Sex	
Female	108 (92)
Male	10 (8)
Race	
African American	22 (19)
White	77 (65)
Other	7 (6)
Multiracial	12 (10)
Ethnicity	
Not Hispanic	104 (88)
Hispanic	14 (12)
Maximum temp (°C) [mean (SD)]	39.3 (1.2)
Fever	
No	12 (10)
Yes	106 (90)
Method of urine collection	
Catheter	56 (47)
Clean catch	62 (53)
Conventional culture result	
Likely UTI^*a*^	74 (63)
Unlikely UTI	23 (19)
No growth	20 (17)
Multiple organisms without a uropathogen^*b*^	3 (2)
Possible UTI	21 (18)
1 uropathogen at 10,000–99,000 CFU/ml with no other growth	5 (4)
2 uropathogens or ≥1 uropathogen along with nonuropathogen(s)	16 (14)

a Likely UTI, growth of a single uropathogen at a count of ≥100,000 CFU/ml without growth of other organisms. UTI, urinary tract infection.

b Diphtheroids, Gram-positive cocci, “multiple organisms.”

**FIG 1:**
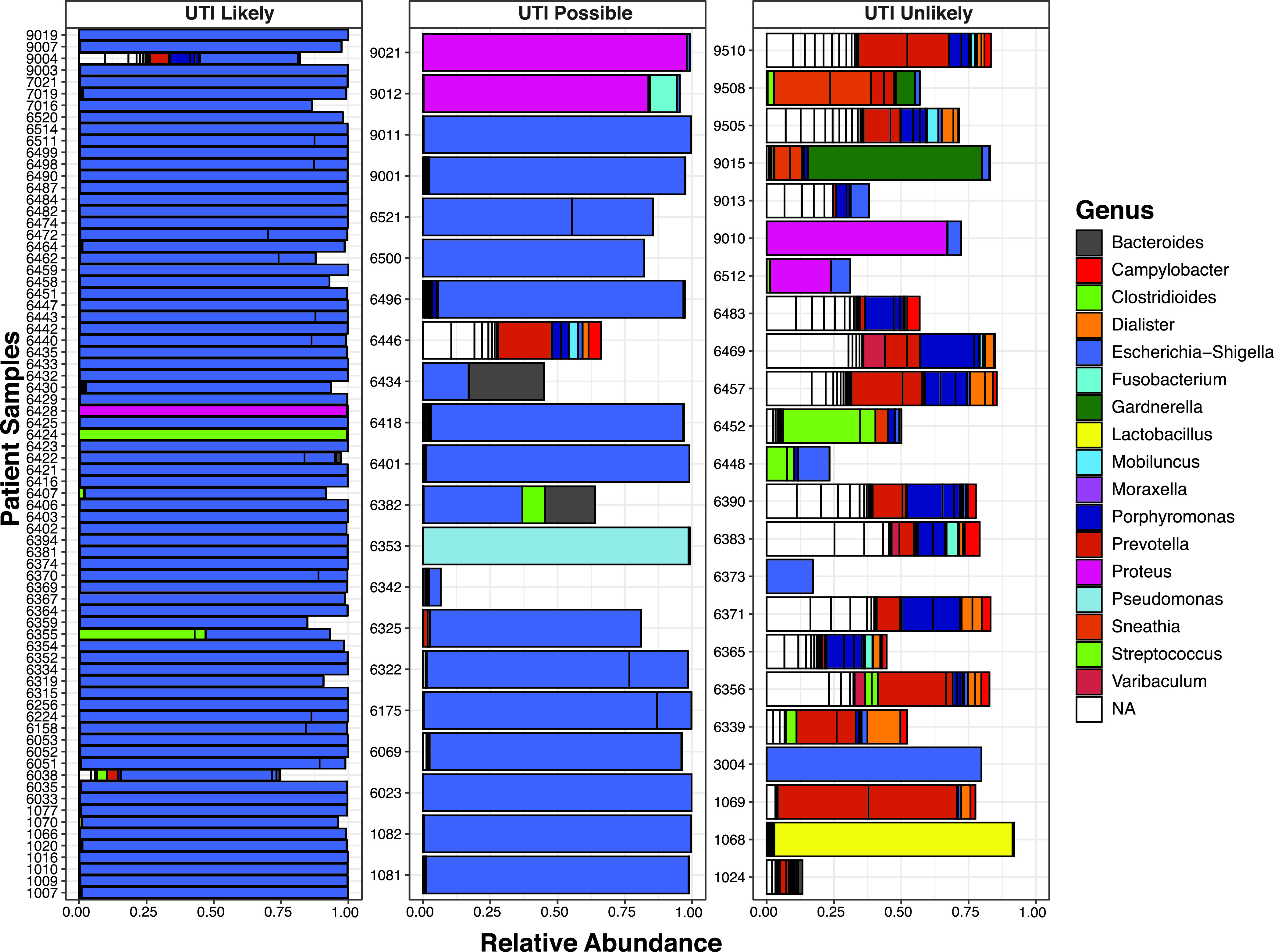
Genus-level taxonomic assignment of the top 50 amplicon sequence variants recovered from urine samples of children with suspected urinary tract infection. Panels are separated by clinical diagnosis according to urine culture results. White bars indicate that no taxonomic assignment was determined. Some bars do not add up to 100% because of sequences that could not be mapped to known taxa. Bars of the same color that are subdivided by vertical lines indicate more than one sequence variant of that organism. Escherichia and Shigella cannot be differentiated at the 16S rRNA gene level, but the clinical context suggests these patient samples contain Escherichia species.

In 70 of the 74 children with likely UTI on conventional culture, defined as ≥100,000 CFU/ml of a single uropathogen, 16S rRNA gene amplicon sequencing analysis confirmed the presence of the same pathogen at the defined criterion of a relative abundance of ≥90% (Table 2, Fig. 1). In the remaining 4 children, while the predominant organism identified by conventional culture and 16S rRNA gene sequencing matched, the relative abundances shown by 16S rRNA gene sequencing were lower than 90%; the clinical and laboratory characteristics of these 4 discrepant cases are shown in Table 3. Of note, 17 of the 72 children with likely UTI and growth of a single type of Escherichia coli on culture had sequences mapping to more than one sequence variant of Escherichia (Fig. 1, bars color coded for Escherichia that are subdivided by vertical lines). A sequence variant of a taxon was defined as a unique 16S rRNA gene sequence, possibly indicating the presence of multiple species or strains.

**TABLE 2:** Agreement between conventional culture and 16S rRNA gene amplicon sequencing

Sample group and UTI diagnosis according to 16S rRNA gene amplicon sequencing^*a*^	No. with indicated UTI diagnosis according to conventional culture^*b*^		
	Likely	Possible	Unlikely
All			
Likely	70	15	0
Possible	2	2	3
Unlikely	2	4	20
Catheter collected			
Likely	46	2	0
Possible	1	1	1
Unlikely	1	1	3
Clean catch collected			
Likely	24	13	0
Possible	1	1	2
Unlikely	1	3	17

a Diagnosis of UTI according to 16S rRNA gene sequencing was categorized as follows: likely UTI was the presence of a uropathogen at ≥90% abundance; possible UTI was the presence of a uropathogen at between 50% and 90%; and unlikely UTI was any sample categorized as neither likely UTI nor possible UTI. UTI, urinary tract infection.

b Diagnosis of UTI according to conventional culture was categorized as follows: likely UTI was growth of a single uropathogen at a count of ≥100,000 CFU/ml without growth of other organisms; possible UTI was growth of a single uropathogen at 10,000 to 99,000 CFU/ml with no other growth, growth of 2 uropathogens, or growth of at least one uropathogen along with nonuropathogen(s); and unlikely UTI was no growth or growth of nonuropathogen(s) in the absence of a uropathogen.

**TABLE 3:** Cases in which results of conventional culture and 16S rRNA gene amplicon sequencing were discordant

Sample	Conventional culture		Age (mo)	Symptom(s)	Method of urine collection	Leukocyte esterase	WBC		16S rRNA gene sequencing	
	Organism(s) and CFU/ml	Interpretation					Count	Unit	Most abundant organism (relative abundance)	Interpretation
6355	E. coli ≥100,000	Likely	6.0	Fever	Catheter	Negative	14	WBC/mm^3^	Escherichia (0.46)	Unlikely
6407	E. coli ≥100,000	Likely	11.4	Fever	Catheter	Moderate (++)	100	WBC/mm^3^	Escherichia (0.897)	Possible
6038	E. coli ≥100,000	Likely	47.6	Fever	Clean catch	Large (+++)	100	WBC/mm^3^	Escherichia (0.61)	Possible
9004	E. coli ≥100,000	Likely	51.0	Urinary symptoms^*a*^	Clean catch	Large (+++)	277	WBC/hpf^*b*^	Escherichia (0.37)	Unlikely
6512	No growth	Unlikely	5.9	Fever	Catheter	Moderate (++)	25	WBC/mm^3^	Enterococcus (0.56)	Possible
3004	No growth	Unlikely	41.5	Urinary symptoms	Clean catch	Small (+)			Escherichia (0.82)	Possible
9010	No growth	Unlikely	96.7	Fever	Clean catch	Large (+++)	62	WBC/hpf	Proteus (0.72)	Possible
6401	E. coli 10,000–50,000	Possible	9.8	Fever	Catheter	Large (+++)	23	WBC/mm^3^	Escherichia (0.99)	Likely
6434	E. coli 10,000–50,000	Possible	8.3	Fever	Catheter	Small (+)	33	WBC/mm^3^	Bacteroides (0.44)^*c*^	Unlikely
9021	Proteus 10,000–50,000	Possible	82.5	Urinary symptoms	Clean catch	Large (+++)	244	WBC/hpf	Proteus (1.00)	Likely
6382	E. coli ≥100,000; Gram-positive cocci ≥100,000	Possible	76.2	Fever	Clean catch	Large (+++)	100	WBC/hpf	Escherichia (0.37)	Unlikely
1081	E. coli ≥100,000; multiple organisms 10,000–50,000	Possible	55.8	Fever	Clean catch	Large (+++)	203	WBC/hpf	Escherichia (0.98)	Likely
6175	E. coli ≥100,000; multiple organisms 10,000–50,000	Possible	93.4	Fever	Clean catch	Large (+++)	135	WBC/hpf	Escherichia (1.00)	Likely
6496	E. coli ≥100,000; multiple organisms 10,000–50,000	Possible	56.4	Fever	Clean catch	Moderate (++)	272	WBC/hpf	Escherichia (0.92)	Likely
6322	E. coli ≥100,000; multiple organisms 10,000–50,000	Possible	57.9	Fever	Clean catch	Large (+++)	100	WBC/hpf	Escherichia (0.98)	Likely
6342	E. coli ≥100,000; multiple organisms 10,000–50,000	Possible	30.8	Fever	Clean catch	Small (+)	25	WBC/mm^3^	Lachnospiraceae (0.42)	Unlikely
6353	Pseudomonas ≥100,000; Klebsiella 10,000–50,000	Possible	8.8	Fever	Catheter	Small (+)	13	WBC/mm^3^	Pseudomonas (0.99)	Likely
1082	E. coli ≥100,000; multiple organisms <10,000	Possible	63.5	Fever	Clean catch	Large (+++)	400	WBC/hpf	Escherichia (0.99)	Likely
6023	E. coli ≥100,000; multiple organisms <10,000	Possible	67.6	Fever	Clean catch	Large (+++)	182	WBC/hpf	Escherichia (1.00)	Likely
6418	E. coli ≥100,000; multiple organisms <10,000	Possible	43.7	Fever	Clean catch	Large (+++)	100	WBC/mm^3^	Escherichia (0.94)	Likely
6500	E. coli ≥100,000; multiple organisms <10,000	Possible	66.2	Fever	Clean catch	Large (+++)	97	WBC/hpf	Escherichia (1.00)	Likely
6521	E. coli ≥100,000; multiple organisms <10,000	Possible	40.1	Fever	Clean catch	Large (+++)	377	WBC/hpf	Escherichia (1.00)	Likely
9001	E. coli ≥100,000; multiple organisms <10,000	Possible	44.2	Urinary symptoms	Clean catch	Moderate (++)	58	WBC/hpf	Escherichia (0.96)	Likely
9011	E. coli ≥100,000; multiple organisms <10,000	Possible	118	Fever	Clean catch	Large (+++)	181	WBC/hpf	Escherichia (1.00)	Likely
6069	E. coli ≥100,000; Gram-positive cocci <10,000	Possible	62.2	Fever	Clean catch	Large (+++)	100	WBC/mm^3^	Escherichia (0. 94)	Likely
6446	E. coli 10,000–50,000; multiple organisms <10,000	Possible	47.4	Fever	Clean catch	Moderate (++)	72	WBC/mm^3^	Ezakiella (0.24)	Unlikely

a Urgency, frequency, dysuria, hesitancy, or incontinence.

b hpf, high-powered field.

c E. coli relative abundance, 25%.

In 20 of the 23 children with unlikely UTI according to the results of conventional culture, defined as no growth or growth of nonuropathogens, 16S rRNA gene sequencing provided concordant results (samples were categorized as neither likely UTI nor possible UTI) (Table 2, Fig. 1); in these 20 children, 16S rRNA gene analysis revealed either many organisms at relative abundances of <50% (*n* = 17) or one predominant (i.e., relative abundance of ≥50%) nonuropathogen (*n* = 3). Discordant cases (*n* = 3) were characterized by the presence of a uropathogen at abundances between 56% and 82% (Table 3); only one had an elevated neutrophil gelatinase-associated lipocalin (NGAL) level.

In 2 of 21 children with possible UTI on conventional culture, defined as 10,000 to 99,000 CFU/ml of a uropathogen or ≥100,000 CFU/ml of a single uropathogen plus other growth, 16S rRNA gene amplicon sequencing analysis provided concordant results (50 to 89% relative abundance of a uropathogen) (Table 2, Fig. 1). Among the 19 discordant cases, 15 were characterized by the presence of a uropathogen at ≥90% abundance.

Focusing on the nonuropathogenic organisms identified by 16S rRNA gene sequencing, a nonuropathogen at a relative abundance of ≥10% was present in a greater proportion of specimens obtained using clean catch than in specimens obtained by catheterization (34% versus 9%, respectively; *P* = 0.002). Specimens from older children were more likely to exhibit nonuropathogens than specimens from younger children (29% in children ≥24 months versus 10% in children <24 months; *P* = 0.02). However, in a multivariate model that included both collection method and age (as a continuous variable), neither variable was significant. No significant differences in the proportions of specimens with nonuropathogens according to sex or race were apparent; however, our sample was too small to allow us to fully explore these factors. The most prevalent nonuropathogens identified by 16S rRNA sequencing were *Ezakiella*, *Prevotella*, and *Porphyromonas*, appearing at abundances of ≥10% in 11%, 10%, and 8%, respectively, of the 118 samples examined.

**FIG 2:**
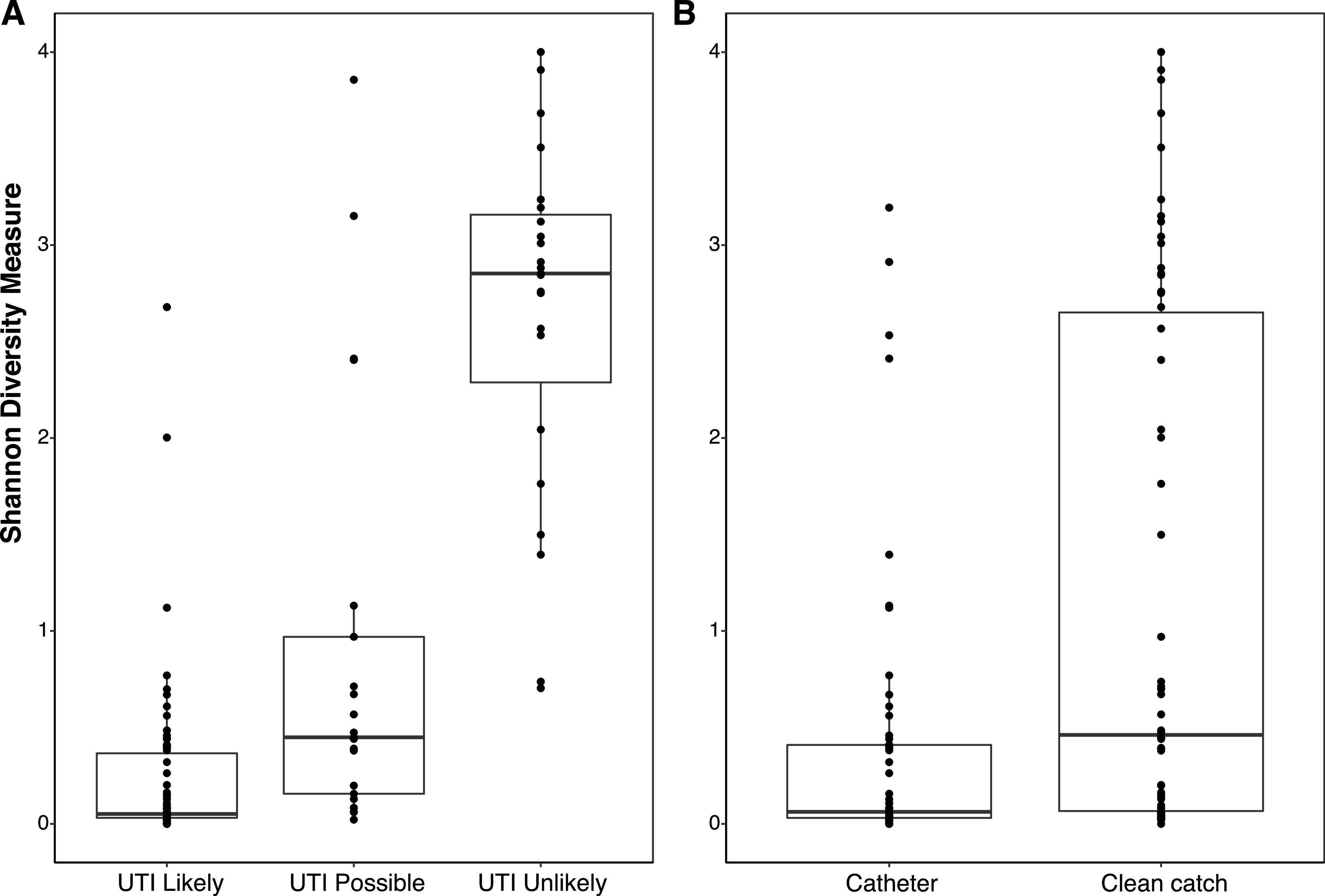
Box plot of Shannon diversity index according to clinical diagnosis (A) and according to method of urine collection (B).

The beta and alpha diversity measures differed significantly in children with unlikely UTI according to the results of their conventional culture. The Bray-Curtis dissimilarity measure of beta diversity indicated a distinct microbial community in samples categorized as unlikely UTI (permutational multivariate analysis of variance [PERMANOVA], *P* = 0.001) (Fig. S1 in the supplemental material). In addition, the Shannon alpha diversity index was significantly higher in the unlikely UTI samples than in the other two groups with clinical UTI diagnoses (Kruskal-Wallis test, *P* < 0.001) (Fig. 2A). Using conventional culture results as the gold standard (with possible and likely UTI categorized as UTI), the area under the curve (AUC) for the Shannon index was 0.91; values of >0.70 are generally considered predictive. A Shannon index of ≤1 had a sensitivity and specificity of 91.6% (95% CI: 86.0 to 97.2%) and 91.3% (95% CI: 79.8 to 100%), respectively, for diagnosing UTI. Samples collected using catheterization had lower alpha diversities than clean catch samples (Mann-Whitney U test, *W* = 1,005.5, *P* < 0.001) (Fig. 2B).

For samples collected using catheterization, the abundance of the predominant pathogen was ≥90% in 48 of 56 samples. In contrast, in clean catch samples, the corresponding proportion of samples was 37 of 62.

## DISCUSSION

This is the largest study to date comparing conventional culture with 16S rRNA gene amplicon sequencing in children with suspected UTI. The concurrent data on urinary markers of inflammation (pyuria) is an added strength.

In children with a positive urine culture result, 16S rRNA gene sequencing and urine culture provided similar results; the tests were concordant in 95% of cases. Furthermore, in discordant cases, the discordance was only a matter of degree and, in our clinical judgement, would not have resulted in changes in clinical management.

In children with an unlikely UTI according to conventional urine culture results, 16S rRNA gene sequencing analysis revealed a uropathogen that was missed by conventional culture in 3 cases (i.e., 13% of children with unlikely UTI and 2.5% of all children in the study). Accordingly, it seems that the use of 16S rRNA gene sequencing analysis would only rarely lead to the identification of UTIs that might be missed by conventional culture. Furthermore, whether these organisms are viable, whether they are numerous or virulent enough to cause significant disease, and whether treating them would lead to more benefit than harm would need to be studied before 16S rRNA gene data could be recommended for use in this capacity. In summary, because we defined pathogens broadly, because we included only children with clinically suspected UTI, because all included children exhibited pyuria, and because we found that the vast majority of children with unlikely UTI according to conventional culture had no uropathogens on 16S rRNA gene amplicon sequencing, our findings, albeit limited, suggest that only a small proportion of children with true UTIs are missed by conventional microbiological techniques.

One aim of this study was to determine whether children with mixed growth would have similarly equivocal results from the 16S rRNA gene analysis. We expected that, given the higher sensitivity of the 16S rRNA gene amplicon sequencing, it would provide results that were even more equivocal. Surprisingly, we found that in children with mixed growth, 16S rRNA gene amplicon sequencing was almost always dominated by a single uropathogen. This suggests that the act of culturing somehow introduces noise that confuses the true signal present at the time of UTI. The reasons for this are not clear. Perhaps the fact that urine is cultured on 3 plates increases the chances of false-positive results. Alternatively, rapid growth under the artificial conditions of the microbiology laboratory of organisms that were relatively low in abundance *in vivo* can introduce noise that confuses the interpretation of urine culture results. Further research in this area is clearly needed.

Overall, concordance between conventional urine culture and 16S rRNA gene amplicon sequencing in this study was high. While colony counts on urine culture are considered absolute and abundance on 16S rRNA gene sequencing is relative, in patients with UTI, 16S rRNA gene results were generally dominated by a single uropathogen at abundances of ≥90%. Thus, albeit relative, abundances in 16S rRNA gene data were rather easy to interpret clinically. Similarly, in most children with unlikely UTI, 16S rRNA gene data most often revealed a variety of organisms previously reported from asymptomatic individuals at relatively low abundances.

16S rRNA gene sequencing is becoming increasingly available at costs approaching that of conventional urine culture. In the near future, we anticipate that sequencing will be able to rapidly identify the uropathogen and perhaps provide information about genes conferring resistance to frequently used antimicrobials. Theoretically, 16S rRNA gene sequencing and analyses of data could occur in a fraction of the time needed for urine culture results to be obtained. The high concordance between the two methods observed in this study would support further investigation into the utility of culture-independent methods of uropathogen detection for management of UTI. At the same time, given the high concordance between the two methods, this study provides reassurance regarding the accuracy of urine culture and provides justification for its continued use until practical challenges regarding the use of 16S rRNA gene amplicon sequencing in clinical settings can be resolved.

In 72 children with likely UTI and a single type of E. coli on conventional culture, we found that 16S rRNA gene sequences mapped to more than one sequence variant of Escherichia in 17 children. Whether this is an indication that more than one type of uropathogen can be involved during a UTI or whether this finding is due to inaccuracies in taxonomic assignment of short sequences is unclear and deserves further study using whole-genome and/or metagenomic sequencing.

Our results extend the findings of previous studies to date on the urobiome of patients with UTI. The study of Ishihara et al. included 10 elderly patients with acute UTI and found that, in 8 cases, 16S rRNA gene and conventional culture results were concordant with regard to the most abundant organism present (7). Kinneman et al. examined the microbiome of 85 children being evaluated for UTI using bladder catheterization (of whom 9 had a UTI according to conventional culture), but detailed data regarding concordance between the two methods are not provided (8).

Using amplicon sequencing, the commensals identified most frequently at abundances of >10% were Ezakiella, Prevotella, and Porphyromonas, which have also been reported in adults. Unlike adults, however, very few children had significant amounts of Lactobacillus and/or Gardnerella (10, 11).

As expected, the average species diversity (as measured by the Shannon index) was lower in children with UTI. We found that diversity alone was a very strong predictor of UTI. Future work is needed to establish the clinical utility of diversity indices in clinical practice.

Our study has several limitations. One goal of using 16S rRNA gene sequencing instead of conventional culture is to detect new pathogens that might have been missed by urine culture, i.e., to improve the reference standard for the diagnosis of UTI. However, at this early stage, it is not clear what organisms should be considered pathogens and what cutoffs (for relative abundance) should be used when interpreting data from 16S rRNA gene sequencing. Regarding the definition of a uropathogen, we used the broadest definition possible to maximize our ability to assess the potential of 16S rRNA gene sequencing to uncover missed UTIs. Accordingly, we categorized any organisms that had previously been reported to cause UTI in earlier studies that used either 16S rRNA gene sequencing or expanded quantitative urine cultures as uropathogens. By defining possible UTI differently, we could have minimized the apparent discordance between the two methods. However, because the aim of the study was to highlight the differences between the two methods, and not to arrive at an exact value summarizing the concordance between the two tests, we decided to retain the *a priori* definition of possible UTI that reflects our clinical practice. Regarding the choice of cutoffs, we selected relative abundance thresholds of 90% and 50% to subdivide 16S rRNA gene results into 3 categories (likely UTI, possible UTI, and unlikely UTI); although these values are arbitrary, the approach used mirrors the pragmatic approach used to diagnose UTI based on conventional culture results. We acknowledge that less abundant organisms may also be capable of causing significant disease. Yet, in order to effectively summarize the available data, we needed to pick cutoffs. Ultimately, the cutoffs chosen had little influence on our conclusions because, in most cases, samples were either dominated by sequences from a single known uropathogen or had very low abundance of a variety of organisms that had, in earlier studies, been detected in urine samples from asymptomatic individuals. Another aspect that could improve the interpretation of these results would be the absolute quantification of each taxon rather than the relative quantification explored here. Methods to quantify the absolute amount of pathogens using quantitative PCR are under development and would be an important improvement for future studies, especially if 16S rRNA gene results were to be used to “rule in” UTI in patients with a negative culture. Our findings directly apply only to symptomatic children being evaluated for a UTI who had pyuria on urinalysis; concordance between the two methods may differ in children with other presentations or in children who lack pyuria. We did not use preservatives before freezing the samples to be used for 16S rRNA gene sequencing; however, samples were never left at room temperature. Only 3 children exhibited growth of multiple organisms unaccompanied by a uropathogen; thus, we are unable to determine whether 16S rRNA gene amplicon sequencing may have been clinically useful in such cases. We acknowledge that, even where both methods reveal the predominance of a uropathogen, this could reflect, in some cases, contamination or colonization.

Future research is needed to clarify the role that 16S rRNA gene or other sequencing approaches can play in the clinical management of children with suspected UTI. These include exploring methods of obtaining estimates of absolute (rather than relative) counts from sequencing analysis, empirically establishing species abundance cutoffs that represent likely UTI, and developing panels that include antibiotic resistance genes.

## MATERIALS AND METHODS

As previously described (12, 13), we prospectively collected urine samples from symptomatic children 2 months to 10 years of age being evaluated at the Children’s Hospital of Pittsburgh, PA, Nationwide Children’s Hospital in Columbus, OH, American Family Children’s Hospital in Madison, WI, Children’s National Health System in Washington, DC, Hasbro Children’s Hospital in Providence, RI, and Primary Children’s Hospital in Salt Lake City, UT, between October 2010 and August 2017 who were clinically suspected of having a UTI (based on the presence of fever and/or symptoms), had a urine sample collected using a method appropriate for their age, had pyuria on urinalysis (defined below), were prescribed antimicrobials for presumed UTI, and had a urine sample available for this study after clinical testing was completed (13). Exclusion criteria included the use of a bag to collect the urine specimen for culture, antibiotic use in the previous 7 days, corticosteroid use in the previous 14 days, chronic disease, genitourinary anomaly, other systemic infectious disease (e.g., pneumonia or sepsis), and immune deficiency. The respective Institutional Review Board at each center approved the study. Consent was obtained at the time of diagnosis. In all cases, samples were collected before the administration of antibiotics for treatment of UTI.

### Conventional urine culture and urinalysis.

Conventional urine culture was performed at the respective microbiological laboratories at the participating institutions using standard microbiological methods. As is customary in clinical care, we categorized the culture results into three categories. Children with growth of a single uropathogen at counts of ≥100,000 CFU/ml were considered as having a “likely UTI.” Taxa previously associated with UTI (Actinobaculum, Aerococcus, Alloscardovia, Citrobacter, Corynebacterium, Enterobacter, Enterococcus, Escherichia, Klebsiella, Morganella, Oligella, Proteus, Pseudomonas, Serratia, Staphylococcus aureus, Staphylococcus lugdunensis, Streptococcus anginosus, and Streptococcus agalactiae) (1) were considered uropathogens. We defined “possible UTI” to include children with a single uropathogen at colony counts between 10,000 and 99,000 CFU/ml with no other growth, children with two uropathogens both at ≥10,000 CFU/ml, and children with at least one uropathogen at ≥10,000 CFU/ml along with nonuropathogen(s). We defined the category of “unlikely UTI” to include children with no growth, children with growth of one or more uropathogens at colony counts of <10,000 CFU/ml, and children with growth of nonuropathogens in the absence of uropathogens. In addition to urine culture, microscopic urinalysis was performed in the clinical laboratories of the respective hospitals. In some hospitals, white blood cell (WBC) counts on microscopy were reported per cubic mm (mm^3^); in other hospitals, results were reported as counts per high-powered field (hpf). Pyuria was defined as ≥10 white blood cells (WBC)/mm^3^, ≥5 WBC/hpf, or ≥1+ leukocyte esterase.

### Urine processing for 16S rRNA gene sequencing.

An aliquot of urine left over after urine culture and urinalysis was complete was used for 16S rRNA gene amplicon sequencing. No preservatives were added, and aliquoting generally occurred within 1 h of collection. However, if delays were anticipated, samples were kept refrigerated. The aliquot was frozen at −80°C until it was shipped on dry ice to the Environmental Sample Preparation and Sequencing Facility at Argonne National Laboratory for 16S rRNA gene amplicon analysis. For DNA extraction, 96-well MO Bio Powersoil DNA (“DNeasy PowerSoil” after acquisition from Qiagen) kits were used (14).

For 16S rRNA gene amplicon sequencing, the V4 region of the 16S rRNA gene (515F-806R) was amplified using PCR with a 12-base barcode on the forward primer (15, 16). The PCR mixture contained 9.5 μl of PCR water, 12.5 μl of Quantabio’s AccuStart II PCR ToughMix (2× concentration, 1× final), 1 μl of Golay barcode-tagged forward primer (5 μM concentration, 200 pM final), 1 μl reverse primer (5 μM concentration, 200 pM final), and 1 μl of template DNA. The PCR conditions were set to 94°C for 3 min to denature the DNA, with 35 cycles at 94°C for 45 s, 50°C for 60 s, and 72°C for 90 s, and a final extension of 10 min at 72°C. Samples were pooled into a single tube, quantified, and diluted to 2 nM, and 6.75 pM of 10% PhiX was added. Amplicons were sequenced on a 151-bp by 12-bp by 151-bp Illumina MiSeq run (16). Negative extraction controls were carried through all the way to sequencing. A positive control of Pseudomonas aeruginosa strain PA14 was included as well; of the 50,059 sequences recovered for this positive control, 49,760 (99.4%) were assigned to Pseudomonas. The remaining sequences in this sample were assigned to a mixture of taxa consisting of Moraxella (0.03%), Parabacteroides (0.06%), Turicibacter (0.02%), and Mucispirillum (0.01%), with the remainder unassigned.

### 16S rRNA gene sequencing analysis.

All samples were demultiplexed using idemp (https://github.com/yhwu/idemp). Demultiplexed files were then imported into R version 3.6.3, and the DADA2 version 1.14.1 pipeline (17) was used to process the sequences. We followed the default settings of the pipeline until the taxonomic classification step, where we used DECIPHER version 2.8.1 (18) and IDTAXA (19). To do this, we used the SILVA small subunit (SSU) r132 March 2018 database and the SILVA species assignment database (20). Samples with fewer than 1,000 sequences were excluded. Following the generation of amplicon sequence variants and taxonomic assignment, we performed additional downstream processing in phyloseq version 1.30.0 (21), which uses vegan version 2.5.6 (22) for diversity calculations and ordinations. For alpha diversity statistics, we used the Mann-Whitney test to determine significant differences between the urine collection methods and the Kruskal-Wallis test followed by the FSA version 0.8.30 (23) implementation of Dunn’s multiple-comparison test using the Benjamini-Hochberg method for testing differences between UTI diagnosis categories. For the ordination, we examined significantly different categories using the adonis implementation of PERMANOVA with a pairwise PERMANOVA *post hoc* test implemented with the RVAideMemoire version 0.9-78 package (24).

We categorized amplicon sequencing results into three categories. “Likely UTI on 16S” was defined by the presence of uropathogens (defined above) at ≥90% relative abundance. The cutoff of 90%, albeit arbitrary, was chosen because it would suggest that organisms in taxa known to cause UTI were present in high abundance in the sample and, as such, would be hard for a clinician to dismiss. “Possible UTI on 16S” was defined as between 50% and 90% relative abundance of a uropathogen. The remainder of the samples were categorized as “unlikely UTI on 16S.”

### Statistical analyses.

We present agreement between conventional culture and 16S rRNA gene analysis by using a 3-by-3 contingency table. For each discrepant case, we provide a list of clinical and laboratory characteristics. Because samples collected using “clean catch” are more likely to be contaminated than samples collected using catheterization, we summarized data according to the method used for urine collection where appropriate. To explore the effects of age, race, sex, and collection method, we used logistic regression to assess whether the proportion of nonuropathogenic taxa differed according to each of these variables.

### Data availability.

All sequences are available under BioProject number PRJNA705267 and BioSample accession numbers SAMN18087111 to SAMN18087230. The R code for sequence processing can be found at https://github.com/sirmicrobe/UTI_manuscript_2021.
