# Supplementary material for: Performance of Conventional Urine Culture Compared to 16S rRNA Gene Amplicon Sequencing in Children with Suspected Urinary Tract Infection
Source: Microbiol Spectr. 2021 Dec 22;9(3):e01861-21. doi: 10.1128/spectrum.01861-21 (PMC8694219; doi:10.1128/spectrum.01861-21)

Supplementary Appendix

Figure S1. Non-metric multidimensional scaling (NMDS) ordination based on the Bray-Curtis dissimilarity measure. Colors indicate clinical diagnosis according to results of clinical culture. Groupings are significantly different ($p<0.001$) based on the adonis implementation of PERMANOVA.

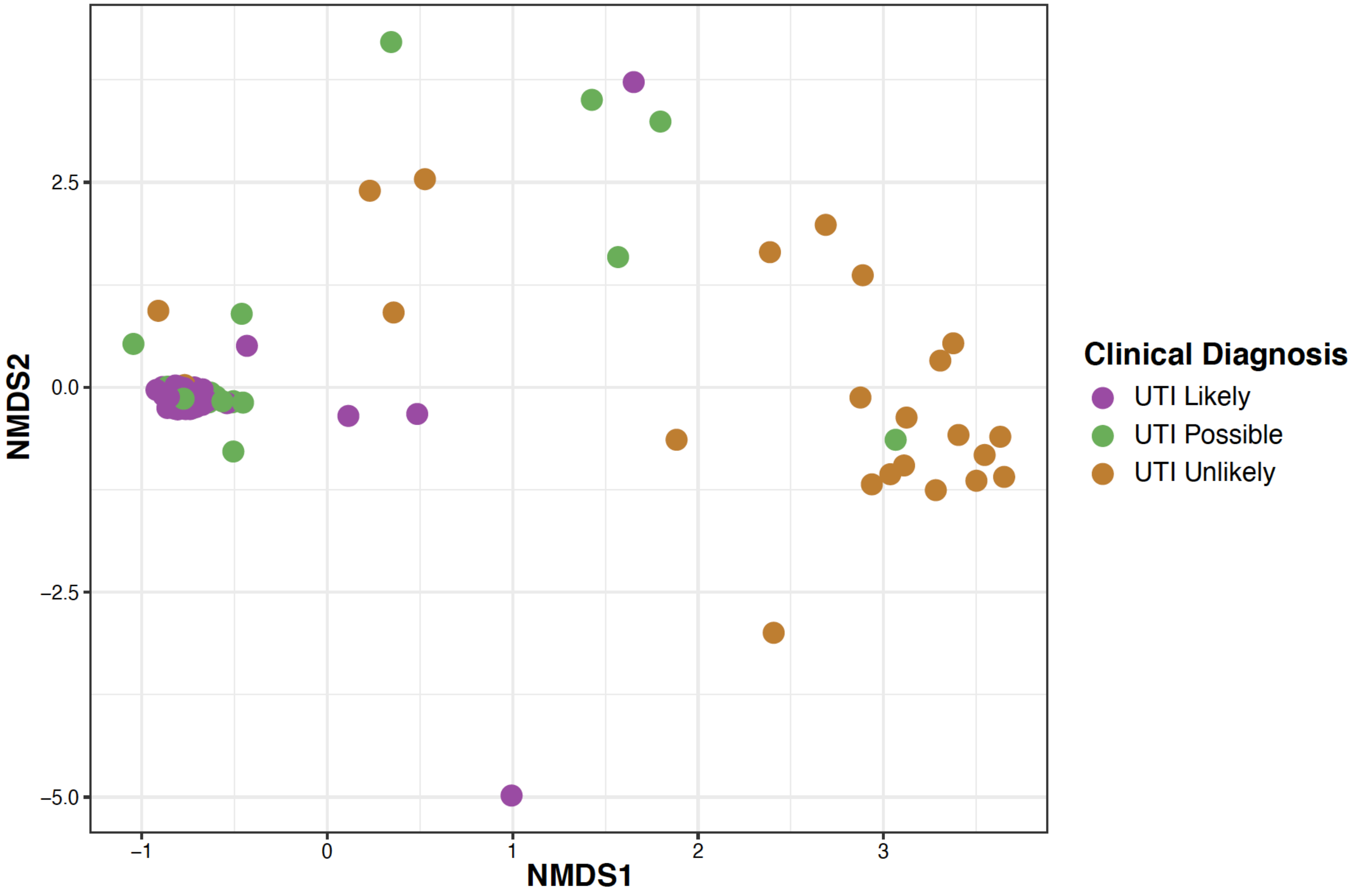

Supplement: SUPPLEMENTAL FILE 1 — Supplemental material. Download SPECTRUM01861-21_Supp_1_seq5.pdf, PDF file, 0.2 MB [file spectrum01861-21_supp_1_seq5.pdf]
